# HIV transmission and retention in care among HIV-exposed children enrolled in Malawi’s prevention of mother-to-child transmission programme

**DOI:** 10.7448/IAS.20.1.21947

**Published:** 2017-09-04

**Authors:** Andreas D. Haas, Joep J. van Oosterhout, Lyson Tenthani, Andreas Jahn, Marcel Zwahlen, Malango T Msukwa, Mary-Ann Davies, Kali Tal, Nozgechi Phiri, Adrian Spoerri, Frank Chimbwandira, Matthias Egger, Olivia Keiser

**Affiliations:** ^a^ Institute of Social and Preventive Medicine (ISPM), University of Bern, Bern, Switzerland; ^b^ Dignitas International, Zomba, Malawi; ^c^ Department of Medicine, College of Medicine, University of Malawi, Blantyre, Malawi; ^d^ International Training & Education Center for Health Malawi, Lilongwe, Malawi; ^e^ Ministry of Health, Lilongwe, Malawi; ^f^ Institute of Global Health, University of Geneva, Geneva, Switzerland; ^g^ Baobab Health Trust, Lilongwe, Malawi; ^h^ Centre of Infectious Disease Epidemiology and Research (CIDER), University of Cape Town, Cape Town, South Africa

**Keywords:** Prevention of mother-to-child transmission (PMTCT), retention, Option B+

## Abstract

**Introduction**: In Malawi, HIV-infected pregnant and breastfeeding women are offered lifelong antiretroviral therapy (ART) regardless of CD4 count or clinical stage (Option B+). Their HIV-exposed children are enrolled in the national prevention of mother-to-child transmission (PMTCT) programme, but many are lost to follow-up. We estimated the cumulative incidence of vertical HIV transmission, taking loss to follow-up into account.

**Methods**: We abstracted data from HIV-exposed children enrolled into care between September 2011 and June 2014 from patient records at 21 health facilities in central and southern Malawi. We used competing risk models to estimate the probability of loss to follow-up, death, ART initiation and discharge, and used pooled logistic regression and inverse probability of censoring weighting to estimate the vertical HIV transmission risk.

**Results**: A total of 11,285 children were included; 9285 (82%) were born to women who initiated ART during pregnancy. At age 30 months, an estimated 57.9% (95% CI 56.6–59.2) of children were lost to follow-up, 0.8% (0.6–1.0) had died, 2.6% (2.3–3.0) initiated ART, 36.5% (35.2–37.9) were discharged HIV-negative and 2.2% (1.5–2.8) continued follow-up. We estimated that 5.3% (95% CI 4.7–5.9) of the children who enrolled were HIV-infected by the age of 30 months, but only about half of these children (2.6%; 95% CI 2.3–2.9) were diagnosed.

**Conclusions**: Confirmed mother-to-child transmission rates were low, but due to poor retention only about half of HIV-infected children were diagnosed. Tracing of children lost to follow-up and HIV testing in outpatient clinics should be scaled up to ensure that all HIV-positive children have access to early ART.

## Introduction

In sub-Saharan Africa, every year 1.4 million children are born to HIV-positive women [1]. Without intervention, 30–45% of the women transmit HIV to their children [2]. With effective antiretroviral therapy (ART) mother-to-child transmission can be reduced to less than 5% [3]. In the last years, huge progress has been made in the global efforts to eliminate HIV mother-to-child transmission [4]. Since 2013, the World Health Organization (WHO) recommends that all HIV-positive pregnant and breastfeeding women are offered lifelong ART according to its “Option B+” guidelines [5]. Most countries with high burden of HIV have implemented Option B+. As a consequence, the ART coverage in pregnant women increased and the number of new HIV infections in children declined [1,6].

Despite progress with prevention of HIV mother-to-child transmission, a substantial number of children are still getting infected [1]. Many of them are born to women who were not diagnosed with HIV, did not initiate ART, discontinued ART or adhered poorly to treatment [7–9]. Infants and young children living with HIV are at a very high risk of mortality: every second untreated HIV-infected child dies before the age of 2 years [10,11]. It is therefore crucial that HIV-positive children are diagnosed and treated soon after infection [10–12]. To ensure timely HIV diagnosis and access to ART, the WHO recommends that children born to HIV-positive women (HIV-exposed children) are regularly followed-up and tested for HIV [5,12]. In Malawi, HIV-exposed children are enrolled in the national prevention of mother-to-child transmission (PMTCT) programme and regularly tested for HIV. Children diagnosed with HIV are referred to the ART clinic and initiate ART [13].

Recent data from the National Evaluation of Malawi’s PMTCT Programme (NEMAPP) showed that mother-to-child transmission antepartum and intrapartum was low with Option B+: 3.9% of the 4–12-week-old children born to HIV-infected mothers were found HIV-positive [14]. While Option B+ successfully prevents antepartum and intrapartum mother-to-child transmission, the policy may prevent postpartum transmission less effectively, because many postpartum women adhere poorly to ART, or discontinue therapy before weaning of breastfeeding [8,9]. Children at higher risk of HIV transmission (i.e. those born to mothers not on ART) are less likely to be retained in care and tested for HIV and thus are underrepresented in routinely collected national data [15–18]. For these reasons, the overall effectiveness of Option B+ for PMTCT at the end of breastfeeding is currently unknown.

We estimated the cumulative incidence of HIV infection at the end of breastfeeding among HIV-exposed children enrolled in Malawi’s PMTCT programme, taking into account unobserved HIV test results from children who were lost to follow-up or not tested. We also estimated cumulative retention of HIV-exposed children in the programme, loss to follow-up, ART initiation and mortality.

## Methods

### Malawi’s mother-to-child transmission programme

In Malawi, HIV-positive pregnant and breastfeeding women are offered lifelong ART according to WHO’s Option B+ policy. HIV-exposed children are enrolled in the national PMTCT programme as soon after birth as possible. Clinic follow-up of children begins at 6 weeks of age. During the first 6 months, children are seen monthly, thereafter every 3 months. Children receive nevirapine prophylaxis for 6 weeks and cotrimoxazole prophylaxis until their final negative HIV status has been confirmed. They are HIV tested with a HIV-1 DNA polymerase chain reaction test at 6 weeks of age, and again with rapid antibody testing when they are 12 and 24 months old. Children are again tested 6 weeks after weaning of breastfeeding to confirm the final negative HIV status before they are discharged. Children diagnosed with HIV are referred for ART initiation [13].

### Study design and participants

**Figure 1.**
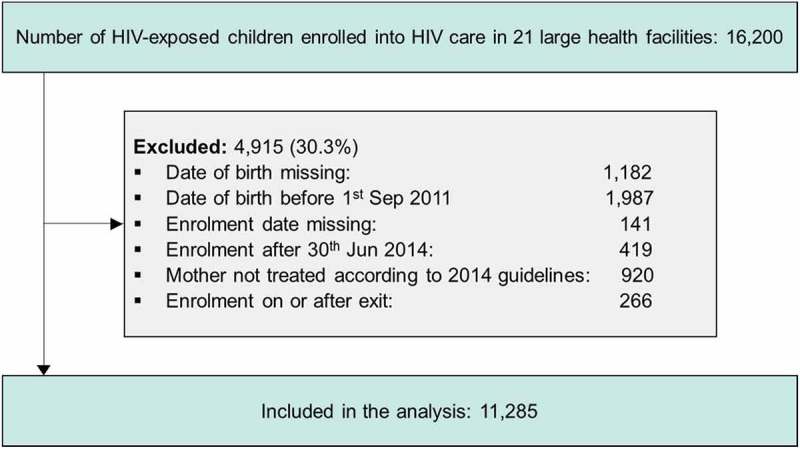
Flow chart of eligibility of study participants.

We included HIV-exposed children who enrolled in Malawi’s national PMTCT programme between 1 September 2011 and 30 June 2014 at one of the 21 large health facilities including health centres, district hospitals, faith-based hospitals and central hospitals from a diverse geographical area in the central and southern region of Malawi who participated in our previous studies of the implementation of Option B+ in Malawi [7–9]. We selected these facilities because they were using the Baobab Health Antiretroviral Therapy (BART) electronic medical record system in September 2011, when the Option B+ programme was launched. Health facilities were classified by the Ministry of Health. Health centres are primary-care facilities, district and faith-based hospitals are secondary-care facilities and central hospitals are tertiary-care facilities. Faith-based hospitals are operated by faith-based organizations. We followed the children up to 26 June 2015. We excluded children whose birthdate was missing, and children born to mothers who received antepartum antiretroviral (ARVs) medication that were no longer recommended in the Malawi’s Integrated HIV Management guidelines (Figure 1) [13].

### Procedures

Registration and follow-up data for HIV-exposed children enrolled in the HIV care programme are routinely collected on standardized paper-based forms kept at the health facilities. Paper-based records were manually captured into an electronic database. To reduce data entry errors, data entry clerks entered the records twice, independently. Inconsistencies were resolved by a third reviewer. Registers were de-duplicated using probabilistic record linkage methods. More details on data collection are given in the appendix (Text S1).

### Outcomes

We analysed the risk of mother-to-child transmission and PMTCT programme outcomes. The endpoints for mother-to-child transmission were the unweighted (observed) cumulative incidence of HIV infection, and the weighted cumulative incidence of HIV infection defined as the sum of the observed cumulative incidence of HIV infection plus the unobserved cumulative incidence of HIV infection in children who were lost to follow-up or not tested for HIV (i.e., the risk of HIV infection that we would have observed if all enrolled children were retained and tested according to the guidelines, after 6 weeks, 12 months and 24 months) [13]. Children were classified as HIV-infected if they tested positive with HIV-1 DNA testing at any time during follow-up, or if their HIV rapid antibody test was positive when they were 12 months or older, or if they were presumptively diagnosed, based on clinical conditions that constitute presumed severe HIV disease and a positive HIV rapid test before they were 12 months old and no later negative test [13]. The endpoints for PMTCT programme outcomes were the estimated proportions of children not yet enrolled into care, retained in care, lost to follow-up, discharged confirmed HIV-free, initiated ART and the proportion of children who died. Children who missed a clinic appointment for more than 60 days and did not return to care thereafter were classified as lost to follow-up. Children who had received a negative HIV test result at least 6 weeks after the end of breastfeeding were discharged confirmed HIV-free. Children were classified as retained in care if they had enrolled into care, and had not been discharged confirmed HIV-free, were not lost to follow-up, had not transferred out, had not initiated ART and had not died.

### Statistical analyses

#### Risk of mother-to-child transmission

We estimated unweighted and weighted Kaplan–Meier failure functions for children’s cumulative incidence of HIV infection in pooled logistic regression. We used inverse probability of censoring weighting to account for HIV infections that would have been observed if all children who enrolled were retained in care and tested according to the guidelines [19,20]. In the weighted analysis, children who were not tested were represented by children who had similar characteristics and were tested. Characteristics considered included infant nevirapine prophylaxis at birth (no, yes, unknown), infant nevirapine prophylaxis after birth (no, yes, unknown), ARVs given to mother during pregnancy (none, mother on triple ART for <4 weeks, mother on triple ART ≥4 weeks, unknown), ARVs given to mother in labour (none, single dose nevirapine, mother on triple ART, unknown), type of facility (faith-based hospital, health centre, district hospital, central hospital), time-updated maternal ART coverage during breastfeeding (on ART, not on ART) and maternal death (yes, no). We calculated approximate 95% confidence intervals (CIs) for the cumulative incidence of HIV infection, using an error factor [21]. We multiplied cumulative incidences by 100 to express cumulative risk of HIV infection as the percentage of HIV-infected children. Text S2 provides more details on the statistical methods we used to estimate the risk of mother-to-child transmission. The analysis was done in STATA (version 14) and data were plotted in R (version 3.2.2).

#### PMTCT programme outcomes

**Figure 2.**
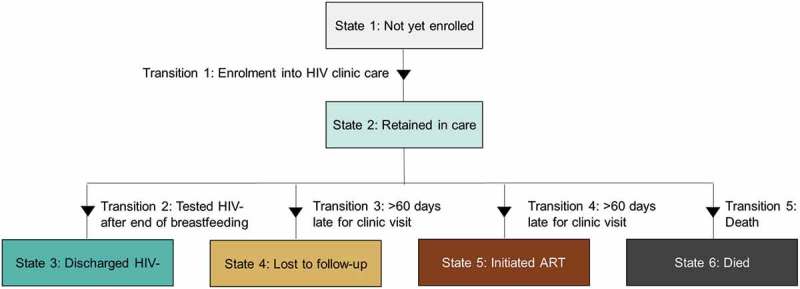
Multi-state model. The boxes represent the six states of the multi-state model and the black triangles represent the events that trigger transitions between states. All children start in State 1 “not yet enrolled” when they are born, and switch to State 2 “retained in care” after they enrolled into HIV care. Children remain in State 2 until they were discharged HIV-negative (State 3), lost to follow-up (State 4), initiated ART (State 5) or died (State 6), or else, until their follow-up ended. States 3 to 6 are absorbing states (i.e. children who have entered an absorbing state remain in this state).

We developed a multi-state model within a competing risk framework to estimate the proportions of children who experienced six PMTCT programme outcomes: enrolment into care, retention in care, loss to follow-up, ART initiation, discharge and death). We fitted the model in the R package mstate [22,23]. The structure of the model is shown in Figure 2. From birth to enrolment into care, children were in State 1 (“not yet enrolled”). On the day they enrolled into the PMTCT programme, children switched to State 2 (“retained in care”), where they remained until they were either discharged confirmed HIV-free (State 3), lost to follow-up (State 4), initiated ART (State 5) or died (State 6). We followed children until the day of discharge, loss to follow-up, death or ART initiation for a maximum follow-up time of 30 months. Children were censored if their follow-up ended, or if they were transferred out. The appendix contains a technical description of the model (Text S2). We compared PMTCT programme outcomes between children at low risk (children who received nevirapine prophylaxis at and after birth and were born to mothers who received at least 4 weeks of triple ART) and high risk (children who received no nevirapine prophylaxis and were born to mothers who received no triple ART) of mother-to-child transmission. We predicted and plotted the percentage of children who had experienced a PMTCT programme outcome separately for children with the low- and high-risk profile. The adjusted hazard ratios (aHRs) from the model we used for the predictions are shown in Table S1.

We fitted a multivariable Cox proportional hazards models in the framework of the multistate model to estimate unadjusted and aHRs for predictors of PMTCT programme outcomes. We considered the following predictors in multivariable analysis: gender (male, female, unknown); age at enrolment (in months); birthweight (≥2500g, <2500g, unknown); nevirapine prophylaxis at birth; nevirapine prophylaxis after birth; antepartum ARV exposure; intrapartum ARV exposure; and, facility type.

### Ethical considerations

The National Health Sciences Research Committee, Malawi and the Cantonal Ethics Committee of Bern, Switzerland granted ethical approval for the study and waived the requirement to obtain informed consent.

## Results

### Characteristics of study participants

We included 11,285 HIV-exposed children from 21 health facilities (Figure 1). Median age at enrolment was 1.7 months (interquartile range (IQR) 1.5–3). Most children (82.3%) were born to women who received ART during pregnancy; 9.6% were born to women who received no antepartum ART. Data on ART exposure during pregnancy were missing for 8.2% of children. Most infants received nevirapine prophylaxis at birth (84.0%) and then continued nevirapine prophylaxis (68.4%) (Table 1).

**Table 1.** Characteristics of children enrolled into HIV care by HIV care outcomes

	**HIV care outcomes**											
	**Retained in care** **(*N* = 4625)**		**Transferred out** **(*N* = 192)**		**Discharged HIV-** **(*N* = 1514)**		**Lost to follow-up** **(*N* = 4662)**		**Died** **(*N* = 72)**		**Initiated ART** **(*N* = 220)**	
**Gender** (%)												
Male	2168	(41.1%)	98	(1.9%)	712	(13.5%)	2169	(41.1%)	32	(0.6%)	97	(1.8%)
Female	2196	(40.4%)	85	(1.6%)	743	(13.7%)	2260	(41.6%)	36	(0.7%)	115	(2.1%)
Unknown	261	(45.5%)	9	(1.6%)	59	(10.3%)	233	(40.6%)	4	(0.7%)	8	(1.4%)
**Age at enrolment** (%)												
<6 weeks	770	(36.2%)	44	(2.1%)	246	(11.6%)	1015	(47.7%)	21	(1.0%)	33	(1.6%)
6–12 weeks	3008	(48.7%)	97	(1.6%)	729	(11.8%)	2236	(36.2%)	30	(0.5%)	75	(1.2%)
3–6 months	523	(32.7%)	30	(1.9%)	257	(16.1%)	742	(46.4%)	11	(0.7%)	37	(2.3%)
>6 months	324	(23.5%)	21	(1.5%)	282	(20.4%)	669	(48.4%)	10	(0.7%)	75	(5.4%)
Median (IQR) age in months	1.6	(1.5–2.3)	1.7	(1.4–3.1)	1.9	(1.5–4.4)	1.8	(1.5–3.5)	1.6	(1.4–3.4)	2.9	(1.6–7.2)
**Year of birth** (%)												
2011	38	(2.8%)	25	(1.8%)	404	(29.5%)	857	(62.5%)	11	(0.8%)	36	(2.6%)
2012	880	(20.2%)	72	(1.7%)	890	(20.4%)	2393	(54.9%)	36	(0.8%)	90	(2.1%)
2013	2893	(63.8%)	80	(1.8%)	212	(4.7%)	1243	(27.4%)	24	(0.5%)	84	(1.9%)
2014	814	(80.0%)	15	(1.5%)	8	(0.8%)	169	(16.6%)	1	(0.1%)	10	(1.0%)
**Birthweight** (%)												
<2.5 kg	641	(41.9%)	28	(1.8%)	190	(12.4%)	611	(39.9%)	24	(1.6%)	37	(2.4%)
≥2.5 kg	2631	(41.4%)	118	(1.9%)	794	(12.5%)	2688	(42.3%)	31	(0.5%)	94	(1.5%)
Missing	1353	(39.8%)	46	(1.4%)	530	(15.6%)	1363	(40.1%)	17	(0.5%)	89	(2.6%)
Median (IQR) in kg	3	(2.7–3.4)	3	(2.7–3.4)	3	(2.7–3.4)	3	(2.7–3.4)	2.8	(2.4–3.2)	3	(2.5–3.3)
**Antepartum ART exposure** (%)												
None	226	(20.9%)	22	(2.0%)	169	(15.6%)	559	(51.8%)	15	(1.4%)	89	(8.2%)
ART <4 weeks	397	(41.4%)	16	(1.7%)	109	(11.4%)	415	(43.3%)	3	(0.3%)	19	(2.0%)
ART ≥4 weeks	3754	(45.1%)	145	(1.7%)	1109	(13.3%)	3171	(38.1%)	48	(0.6%)	99	(1.2%)
Unknown	248	(27.0%)	9	(1.0%)	127	(13.8%)	517	(56.2%)	6	(0.7%)	13	(1.4%)
**Intrapartum ARV exposure** (%)												
None	290	(23.4%)	27	(2.2%)	221	(17.8%)	595	(48.0%)	16	(1.3%)	91	(7.3%)
sdNVP	113	(33.0%)	4	(1.2%)	63	(18.4%)	152	(44.4%)	5	(1.5%)	5	(1.5%)
ART	3935	(45.1%)	147	(1.7%)	1110	(12.7%)	3380	(38.7%)	47	(0.5%)	114	(1.3%)
Unknown	287	(29.6%)	14	(1.4%)	120	(12.4%)	535	(55.2%)	4	(0.4%)	10	(1.0%)
**NVP at birth** (%)												
None	302	(25.7%)	24	(2.0%)	213	(18.1%)	534	(45.4%)	12	(1.0%)	90	(7.7%)
Yes	4151	(43.8%)	160	(1.7%)	1226	(12.9%)	3773	(39.8%)	56	(0.6%)	119	(1.3%)
Unknown	172	(27.5%)	8	(1.3%)	75	(12.0%)	355	(56.8%)	4	(0.6%)	11	(1.8%)
**NVP after birth** (%)												
None	616	(31.2%)	30	(1.5%)	355	(18.0%)	854	(43.3%)	16	(0.8%)	103	(5.2%)
Yes	3487	(45.2%)	139	(1.8%)	978	(12.7%)	2980	(38.6%)	40	(0.5%)	96	(1.2%)
Unknown	522	(32.8%)	23	(1.4%)	181	(11.4%)	828	(52.0%)	16	(1.0%)	21	(1.3%)
**Facility type (%)**												
Health centre	684	(48.5%)	11	(0.8%)	59	(4.2%)	639	(45.3%)	0	(0.0%)	18	(1.3%)
District hospital	3224	(41.4%)	119	(1.5%)	1220	(15.7%)	3010	(38.7%)	47	(0.6%)	162	(2.1%)
Faith-based hospital	397	(39.2%)	20	(2.0%)	117	(11.5%)	442	(43.6%)	12	(1.2%)	26	(2.6%)
Central hospital	320	(29.7%)	42	(3.9%)	118	(10.9%)	571	(53.0%)	13	(1.2%)	14	(1.3%)

Data are number of children (row %) if not otherwise stated. ARV: antiretroviral drugs; ART: antiretroviral therapy; NVP: nevirapine prophylaxis.

### Risk of mother-to-child transmission

**Figure 3.**
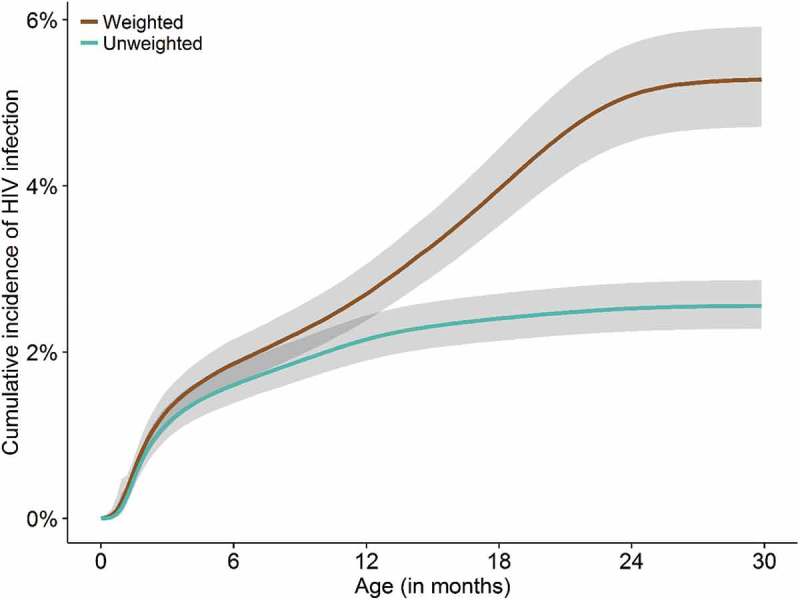
Unweighted and weighed cumulative incidence of HIV infection in HIV-exposed children. Estimates of the cumulative incidence of HIV infection among all children enrolled into care from unweighted and weighted analyses. The weighted analyses correct for unobserved test results in children lost to follow-up or not tested Shaded areas show 95% confidence intervals.

Overall, 288 out of 11,285 children (2.6%) were diagnosed with HIV. Most children (272, 94.4%) were diagnosed based on a positive HIV-1 DNA or a positive HIV rapid antibody test taken after the age of 12 months. Few children (16 of 288, 5.6%) were diagnosed as presumed severe HIV disease. Figure 3 shows the estimated cumulative incidence of HIV infection from unweighted and weighted analyses. By age 8 weeks, 0.7% (95% CI: 0.6–0.9) of the children who enrolled were diagnosed as HIV infected. The proportion of children who were diagnosed increased to 2.2% (95% CI: 1.9–2.5) by age 12 months and to 2.6% (95% CI: 2.3–2.9) by age 30 months. In the weighted analysis, which takes unobserved test results from children lost to follow-up or not tested into account, the cumulative incidence was 0.8% (95% CI: 0.7–1.0) by age 8 weeks, 2.7% (95% CI: 2.4–3.1) by age 12 months and 5.3% (95% CI: 4.7–5.9) by age 30 months.

### PMTCT programme outcomes

**Figure 4.**
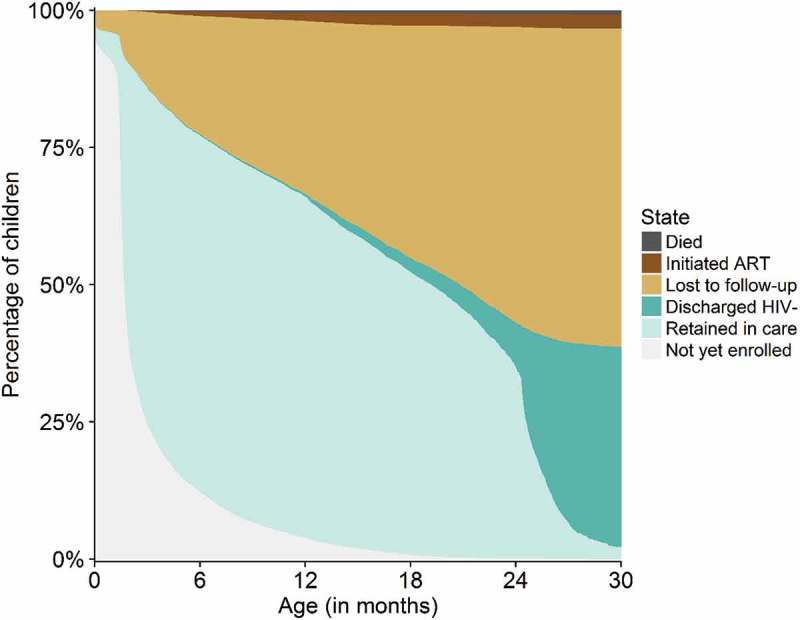
Percentages of HIV-exposed children not yet enrolled, retained in care, discharged HIV-negative, lost to follow-up, initiated ART or dead. The coloured areas show the percentage of children in the corresponding state at the given time. Children were discharged confirmed HIV-free if they were tested HIV-negative at least 6 weeks after cease of breastfeeding. Children were considered lost to follow-up if they had missed a clinic appointment for more than 60 days and did not return to care thereafter.

Figure 4 shows the proportions of HIV-exposed children who were not yet enrolled into care, retained in care, lost to follow-up, discharged HIV-negative, initiated ART or who died within the first 30 months of their lives. By the age of 6 months, 12.2% of the children (95% CI: 11.6–12.8) were not yet enrolled into care. Only few children (3.8%; 95% CI: 3.5–4.2) enrolled after age 12 months. By the age of 6 months, 21.5% (95% CI: 20.7–22.3) of the children were lost to follow-up; this number increased to 31.5% (95% CI: 30.6–32.4) by 12 months, and 57.9% (95% CI: 56.6–59.2) by 30 months. Documented mortality was low: 0.8% (95% CI: 0.6–1.0) of the children were known to have died by the age of 30 months. Most children who were discharged HIV-free received their final negative HIV test result between 24 and 30 months. By age 30 months, 36.5% (95% CI: 35.2–37.9) of the children were discharged HIV-negative, 2.6% (95% CI: 2.3–3.0) initiated ART and 2.2% (95% CI: 1.5–2.8%) were followed beyond age 30 months.

Table 2 shows the results from the multivariable analysis of predictors of PMTCT programme outcomes. Results from univariable analyses are shown in the appendix in Table S2. Children who enrolled late, those who received care at health centres and those born to mothers who did not receive ART during pregnancy were at the highest risk of loss to follow-up. The aHRs for loss to follow-up was 1.13 (95% CI: 1.12–1.14) per month increase in the age at enrolment of the child. Children born to women who received ART during pregnancy were much less likely to be lost to follow-up than children of women who received no ART during pregnancy: for women who received ART <4 weeks, the aHR of loss to follow-up was 0.77 (95% CI: 0.65–0.91), and for women who received ART for ≥4 weeks, the aHR was 0.62 (95% CI: 0.54–0.72). The risk of loss to follow-up was highest in central hospitals, followed by health centres, and faith-based hospitals, and lowest in district hospitals. Children who received nevirapine prophylaxis, and those born to women who received ART during pregnancy, enrolled at younger ages than those who were not exposed to ARV medication. Low birthweight (<2.500g) and no antepartum ART exposure was associated with ART initiation. Later enrolment into care and low birthweight was associated with increased mortality.

**Table 2.** Multivariable analysis of predictors of time to enrolment, loss to follow-up, discharged HIV-negative, ART initiation and mortality

	Enrolment		Loss to follow-up		Discharge HIV-		ART initiation		Mortality	
	aHR	95% CI	aHR	95% CI	aHR	95% CI	aHR	95% CI	aHR	95% CI
**Gender**										
Male	1		1		1		1		1	
Female	0.99	(0.95–1.03)	1.02	(0.96–1.08)	0.97	(0.87–1.08)	1.16	(0.88–1.53)	0.88	(0.53–1.48)
Unknown	0.98	(0.89–1.06)	1.13	(0.99–1.29)	1.12	(0.84–1.49)	0.76	(0.35–1.64)	1.37	(0.48–3.94)
**Age at enrolment (months)**	NA		1.13	(1.12–1.14)	1.00	(0.99–1.01)	1.23	(1.18–1.29)	1.12	(1.02–1.23)
**Birthweight**										
≥2.5 kg	1		1		1		1		1	
<2.5 kg	0.96	(0.91–1.01)	0.98	(0.89–1.07)	0.88	(0.74–1.05)	1.71	(1.17–2.52)	2.72	(1.52–4.85)
Missing	0.70	(0.67–0.74)	0.95	(0.89–1.02)	0.99	(0.88–1.13)	1.14	(0.83–1.55)	0.73	(0.38–1.41)
**Antepartum ARV exposure**										
None	1		1		1		1		1	
ART <4 weeks	1.14	(1.01–1.29)	0.77	(0.65–0.91)	0.93	(0.68–1.26)	0.45	(0.22–0.94)	0.22	(0.05–1.01)
ART ≥4 weeks	1.23	(1.10–1.37)	0.62	(0.54–0.72)	1.08	(0.84–1.38)	0.25	(0.13–0.48)	0.36	(0.12–1.11)
Unknown	1.14	(0.99–1.31)	0.84	(0.69–1.02)	0.84	(0.58–1.22)	0.45	(0.18–1.14)	0.46	(0.09–2.47)
**Intrapartum ARV exposure**										
None	1		1		1		1		1	
sdNVP	1.01	(0.88–1.16)	1.11	(0.91–1.35)	0.97	(0.70–1.34)	0.57	(0.22–1.48)	1.21	(0.34–4.31)
ART	1.03	(0.92–1.14)	1.06	(0.92–1.23)	0.85	(0.68–1.07)	0.85	(0.45–1.60)	0.76	(0.24–2.39)
Unknown	1.08	(0.94–1.24)	1.20	(0.99–1.45)	1.01	(0.70–1.46)	0.33	(0.11–1.03)	0.21	(0.03–1.42)
**NVP at birth**										
None	1		1		1		1		1	
Yes	1.46	(1.33–1.59)	0.93	(0.83–1.06)	1.04	(0.85–1.28)	0.46	(0.29–0.75)	0.75	(0.30–1.88)
Unknown	1.07	(0.93–1.23)	1.02	(0.84–1.23)	1.27	(0.87–1.85)	0.95	(0.38–2.41)	1.06	(0.23–4.93)
**NVP after birth**										
None	1		1		1		1		1	
Yes	1.28	(1.20–1.36)	0.95	(0.86–1.05)	1.04	(0.88–1.23)	0.83	(0.53–1.29)	0.93	(0.41–2.08)
Unknown	1.22	(1.12–1.32)	1.24	(1.10–1.40)	0.86	(0.69–1.08)	0.82	(0.45–1.49)	2.80	(1.15–6.80)
**Facility type**										
Faith-based Hospital	1		1		1		1		1	
Health Centre	1.14	(1.05–1.24)	1.38	(1.22–1.56)	0.83	(0.56–1.23)	0.79	(0.43–1.47)	0.00	(0.00->100)
District Hospital	1.33	(1.25–1.42)	0.83	(0.75–0.92)	1.56	(1.27–1.92)	0.84	(0.54–1.30)	0.45	(0.23–0.87)
Central Hospital	1.59	(1.46–1.73)	1.22	(1.08–1.39)	2.33	(1.76–3.08)	0.77	(0.39–1.49)	0.87	(0.37–2.07)

aHR: adjusted hazards ratios; CI: confidence interval; ARV: antiretroviral drugs; ART: antiretroviral therapy; NVP: nevirapine prophylaxis.

**Figure 5.**
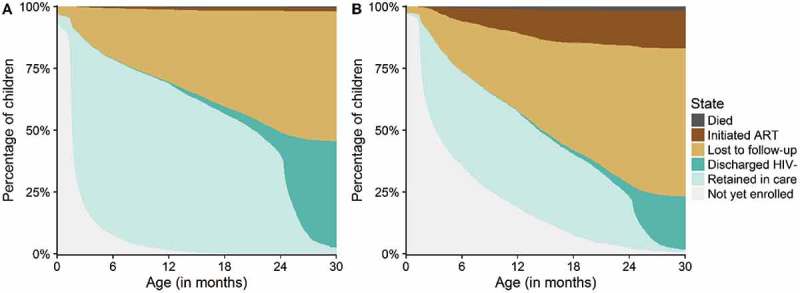
Percentages not yet enrolled, retained in care, discharged HIV-negative, lost to follow-up, initiated ART or dead among HIV-exposed children at low and at high risk of mother-to-child transmission. Figure (a) (low-risk profile) shows HIV care outcomes for children who received nevirapine prophylaxis at and after birth and who were born to women who received antiretroviral therapy (ART) during and after pregnancy. Figure (b) (high-risk profile) shows HIV care outcomes of children who received no nevirapine prophylaxis and were born to women who were not on ART.

Figure 5 shows the proportions of children not yet enrolled, retained in care, discharged confirmed HIV-free, lost to follow-up, initiated ART or dead, for children at low and high risk of HIV mother-to-child transmission. Children at high risk of mother-to-child transmission enrolled later into care, were more likely to be lost to follow-up, more likely to die, much more likely to initiate ART and less likely to be discharged HIV-negative than children at low risk of mother-to-child transmission.

## Discussion

We estimated that just over 5% of the HIV-exposed children enrolled in Malawi’s PMTCT programme were HIV-infected by the age of 30 months, but due to high loss to follow-up only about half of these children were diagnosed in the programme. About 80% of children received nevirapine prophylaxis and were born to women who received ART, but retention of children in the PMTCT programme was poor: almost two thirds were lost to follow-up and only one third of the children were discharged confirmed HIV-negative. Children who enrolled late, those who did not receive nevirapine prophylaxis and those who were born to women who did not receive ART during pregnancy were at highest risk of loss to follow-up.

Our study is the first to estimate the risk of mother-to-child transmission at the end of breastfeeding in Malawi’s Option B+ programme. The median duration of breastfeeding in Malawi is 23 months and only few children were still breast-fed beyond our follow-up duration [24]. A strength of our study is that we adjusted our estimates for HIV transmission to take into account unobserved HIV test results from children lost to follow-up or not tested, and that we thus could estimate the cumulative incidence of HIV infection for all children enrolled in the PMTCT programme [19,20]. Our study also has several limitations. We did not account for the unobserved risk in unknown HIV-exposed children, that is, children born to HIV-positive women whose HIV infection was not diagnosed during pregnancy or breastfeeding [7]. Hence our study may slightly underestimate the risk of mother-to-child transmission in the Option B+ programme. We collected data from 21 large health centres, district hospitals, faith-based hospitals and central hospitals from a diverse geographical area in the central and southern region of Malawi, but we did not include data from the northern region, or from smaller rural health centres. Our findings thus may not be representative for all health facilities in Malawi.

The recent nation-wide evaluation of Malawi’s PMTCT Programme has shown that Option B+ effectively prevents antepartum and intrapartum transmission [14]; however, the Option B+ policy may prevent postpartum transmission less effectively, because many postpartum women adhere poorly to ART, or discontinue therapy before weaning of breastfeeding [8,9]. Two studies from Malawi have described the risk of postpartum HIV transmission among HIV-exposed children who were retained in care and tested for HIV [25,26]. A cohort study from a large urban district hospital in Malawi’s capital Lilongwe showed that 6.2% of the enrolled children were diagnosed with HIV infection by age 24 months. Almost half of the children were lost to follow-up and HIV testing rates at the end of breastfeeding were low [25]. A study from Thyolo district hospital in the Southern region of Malawi reported that 4.1% of the HIV-exposed children enrolled in the PMTCT programme were diagnosed as HIV infected at 12 months of age. Two thirds of the children were retained, but less than half of the children were tested for HIV at 12 months [26]. Neither of the two studies adjusted for missing data in children who were lost to follow-up. As children at higher risk of mother-to-child transmission are underrepresented in routine PMTCT programmes, both studies will have underestimated the risk of mother-to-child-transmission in the Option B+ programme [15–18]. The present analysis shows that the degree of underestimation can be substantial. Of note, the unweighted mother-to-child transmission rates in both studies were higher than our estimates. This may be explained by differences in retention rates among mothers on ART at different facilities and differences in implementation of tracing programmes to bring children lost to follow-up back into care [25,27].

Malawi has made huge progress in its efforts to eliminate HIV mother-to-child transmission. Malawi was the first country to offer lifelong ART to all HIV-positive pregnant and breastfeeding women under its Option B+ guidelines [13]. After Option B+ was implemented, the ART coverage among pregnant women quickly rose: up to 80% of the HIV-positive women in Malawi received ART during pregnancy [1]. In our study, 82% of children were born to women who initiated ART during pregnancy, confirming the national estimates. The high ART coverage has led to low antepartum and intrapartum mother-to-child transmission rates. Our study shows that the overall transmission risk under Option B+ at the end of breastfeeding was still low: an estimated 5.3% of the children were infected by age 30 months. Our data suggest that Option B+ as implemented in Malawi effectively lowers the risk of antepartum, intrapartum and postpartum HIV transmission.

We estimated that every second HIV-infected child remains undiagnosed within the PMTCT programme because retention in care is inadequate and uptake of HIV testing is poor, especially among children born to women who were not on ART. In line with previous studies, we showed that children born to women who received ART are less likely to be lost to follow-up and more likely to be tested for HIV [15–18]. Efforts to increase retention and HIV testing could improve the diagnosis of HIV-infected children and their access to ART. Better retention could also improve poor health outcomes among HIV-exposed uninfected children [28]. Interventions to improve retention should address possible drivers of loss to follow-up, including non-disclosure of HIV status, low socio-economic status of parents, low maternal education, lack of support from family members and partners and fear of stigma [29–31]. An integrated infant tracing programme in which community health workers traced HIV-exposed children was highly successful: 77% of the defaulters could be reached by phone or home visit, and 95% of them returned to care [32]. A similar tracing intervention at a large district hospital in Lilongwe was less successful: only half of the children could be traced and only one-third of those who were located could be returned to care [25]. Conditional cash transfers or transport reimbursements are other possible interventions to improve retention in care [33]. HIV testing within PMTCT programmes could be improved by addressing logistical challenges such as shortages of dried blood spot sample test kits [34]. Additionally, HIV testing could be performed in paediatric in-patient and out-patient clinics to ensure access to ART of HIV-positive children who dropped out of PMTCT programmes [35].

## Conclusions

Confirmed HIV mother-to-child transmission rates in Malawi’s Option B+ programme are low, but due to poor retention, less than half of the HIV-infected children were diagnosed. We recommend expansion of tracing of children lost to follow-up and HIV testing in outpatient clinics to ensure that all HIV-positive children have access to early ART.
